# Supplementary material for: The genome sequence of a leaf beetle, Galerucella nymphaeae (Linnaeus, 1758)
Source: Wellcome Open Res. 2025 Feb 10;10:57. [Version 1] doi: 10.12688/wellcomeopenres.23676.1 (PMC11880761; doi:10.12688/wellcomeopenres.23676.1)
Supplement: Peer review history

# Reviewer report: Reviewer response for version 1

**DOI:** 10.21956/wellcomeopenres.26119.r119561

**Published:** 2025-07-07

**Authors:** Hans-Peter Fuehrer

**Affiliations:** 1 University of Veterinary Medicine, Vienna, Austria

The authors clearly describe the genome sequence analysis of *Galerucella nymphaeae*.

 Please provide proper information how the specimen was collected.

Are sufficient details of methods and materials provided to allow replication by others?

Yes

Is the rationale for creating the dataset(s) clearly described?

Yes

Are the datasets clearly presented in a useable and accessible format?

Yes

Are the protocols appropriate and is the work technically sound?

Yes

Reviewer Expertise:

medical entomology and barcoding

I confirm that I have read this submission and believe that I have an appropriate level of expertise to confirm that it is of an acceptable scientific standard.

# Reviewer report: Reviewer response for version 1

**DOI:** 10.21956/wellcomeopenres.26119.r118909

**Published:** 2025-03-04

**Authors:** Aureliano Bombarely

**Affiliations:** 1 Institute for Molecular and Cellular Plant Biology (IBMCP, CSIC-UPV), Valencia, Spain

**Summary of the key results**

 The manuscript titled “The genome sequence of a leaf beetle, *Galerucella nymphaeae* (Linnaeus, 1758)” describes the genome sequencing, and assembly of the beetle species *Galerucella nymphaeae*. The assembly size was 350 Mb in 32 scaffolds. 99.8% of the assembly was anchored into the 16 expected pseudomolecules, including the X chromosome. The completeness was evaluated with BUSCO (99.1%) and Merqury (98.88%).

**Overall evaluation**

 The assembly metrics looks fine. The methodology has been described in detail. There is not any concern about the presentation of the data. Only a minor suggestion, that it will be to link the species to a database like GBIF (e.g., https://www.gbif.org/species/1048344) for the user to retrieve more information if needed.

**Discretionary changes**

- **No information about the second haplotype was supplied. **The manuscript text mentions “Contigs corresponding to the second haplotype have also been deposited” but no stats or any other information was supplied.

Are sufficient details of methods and materials provided to allow replication by others?

Yes

Is the rationale for creating the dataset(s) clearly described?

Yes

Are the datasets clearly presented in a useable and accessible format?

Yes

Are the protocols appropriate and is the work technically sound?

Yes

Reviewer Expertise:

Evolutionary Genomics, Genome Assembly and Annotations (focused in plants)

I confirm that I have read this submission and believe that I have an appropriate level of expertise to confirm that it is of an acceptable scientific standard.

# Reviewer report: Reviewer response for version 1

**DOI:** 10.21956/wellcomeopenres.26119.r118496

**Published:** 2025-03-04

**Authors:** Badr M Al-Shomrani

**Affiliations:** 1 King Abdulaziz City for Science and Technology, Riyadh, Saudi Arabia

The researchers sequenced the genome of a female *Galerucella nymphaeae* specimen using Hi-Fi and Hi-C technologies, generating 2.43 million and 689.35 million reads, respectively. The final assembly demonstrated high quality, with a BUSCO score of 99.1%, a Quality Value of 64.4, and a k-mer completeness of 99.98% for the combined assemblies. The methodology was clearly described, and the assembly appears robust.

Are sufficient details of methods and materials provided to allow replication by others?

Yes

Is the rationale for creating the dataset(s) clearly described?

Yes

Are the datasets clearly presented in a useable and accessible format?

Yes

Are the protocols appropriate and is the work technically sound?

Yes

Reviewer Expertise:

Bioinformatics

I confirm that I have read this submission and believe that I have an appropriate level of expertise to confirm that it is of an acceptable scientific standard.
